# A hardware-grounded energy taxonomy for comparing deep and Spiking Neural Network inference on edge platforms

**DOI:** 10.3389/fnins.2026.1919565

**Published:** 2026-08-11

**Authors:** Mohamed El-Hafci, My Abdelouahed Sabri, Abdellah Aarab

**Affiliations:** Laboratory of Engineering, Modeling and Systems Analysis (LIMAS), Faculty of Sciences Dhar El Mahraz (FSDM), Sidi Mohamed Ben Abdellah University, Fez, Morocco

**Keywords:** CMOS power modeling, edge AI accelerators, energy efficiency taxonomy, HW/SW co-design, neuromorphic engineering, Spiking Neural Networks

## Abstract

Energy consumption is becoming one of the main constraints for neural network inference on edge devices, where compute, memory, and power budgets are tight. A large number of works already study the efficiency of Deep Neural Networks (DNNs), and a growing body of work does the same for Spiking Neural Networks (SNNs). However, comparing the two on equal terms is not straightforward because existing analyses rarely rely on a shared decomposition of inference energy linked to the actual hardware behavior. This Mini Review synthesizes hardware-aware studies into a unified taxonomy that decomposes inference energy into seven contributions: computation *E*_*compute*_, memory access *E*_*memory*_, internal state *E*_*state*_, temporal processing *E*_*temporal*_, activation *E*_*activation*_, static leakage *E*_*leakage*_, and clock distribution *E*_*clock*_. The corresponding expressions are derived from classical CMOS energy models and interpreted using representative findings from hardware studies. We illustrate the taxonomy using representative studies on microcontrollers, FPGAs, ASICs, and neuromorphic processors. The objective is not to recommend one paradigm. It is to provide a reading grid close to the hardware that can support architectural choices when energy, accuracy, and latency cannot all be optimized at once.

## 1 Introduction

The deployment of neural network inference executed on energy- and resource-limited edge platforms has become a major challenge of embedded artificial intelligence, where energy, memory, and computational resources are severely constrained (Ngo et al., 2025). Under these constraints, energy becomes a primary architectural concern that cannot be fully captured by high-level proxies such as FLOPs or parameter counts alone and therefore requires hardware-level analysis (Chandrakasan and Brodersen, 1995; Horowitz, 2014). This challenge is exacerbated by the breakdown of Dennard scaling, which once dictated that shrinking conventional transistors would automatically reduce power, thereby forcing edge platforms to rely on microarchitectural innovations such as execution sparsity and hardware-algorithm co-design (Sze et al., 2017; Hennessy and Patterson, 2019). In the context of neural network inference, these hardware-level constraints manifest across multiple architectural layers–from arithmetic units performing multiply-accumulate (MAC) operations, to the memory hierarchy storing weights and activations, to the clock distribution network–with memory access costs consistently identified as the dominant bottleneck.

Two fundamentally distinct neural network paradigms are considered for edge deployment. Conventional Deep Neural Networks (DNNs) operate through dense, synchronous, real-valued matrix multiplications, with energy profiles dominated by MAC operations and weight-memory access patterns (Yoon et al., 2025). Conversely, Spiking Neural Networks (SNNs) utilize sparse binary spike trains and accumulate-only (AC) operations, leveraging event-driven sparsity to mitigate computational and communication overheads (Davidson and Furber, 2021; Rathi et al., 2023). Despite widespread claims of inherent SNN energy efficiency, comparative studies frequently rely on hardware-agnostic metrics that omit memory access costs, state maintenance overhead, and temporal processing penalties–leading to potentially misleading conclusions (Dampfhoffer et al., 2023; Ferreira et al., 2025).

This Mini Review synthesizes hardware-aware studies into a unified taxonomy for DNN and SNN inference. Unlike previous works focusing on specific energy components, the proposed taxonomy provides a unified framework for interpreting published energy analyses. The objective is not to introduce a new energy estimation methodology, but to provide a structured perspective for consistently interpreting energy trade-offs across edge AI hardware.

## 2 Physical foundations and energy taxonomy

Energy dissipation in CMOS-based digital platforms–microcontrollers, FPGAs, ASICs, or neuromorphic processors–arises from three mechanisms: dynamic switching power from capacitance charge/discharge at logic transitions $P_{d\,y\,n\,a\,m\,i\,c}=\alpha \times C\times V_{D\,D}^{2}\times f$; static leakage through sub-threshold and gate-oxide currents; and transient short-circuit power during transitions (Chandrakasan and Brodersen, 1995; Ng et al., 2022). In neural network inference, these mechanisms contribute non-uniformly. Horowitz (2014) established that in 45 nm CMOS, a single off-chip DRAM access (∼640 pJ) exceeds an 8-bit multiplication (∼0.2 pJ) by three orders of magnitude, positioning data movement, not arithmetic, as the primary energy bottleneck (Davidson and Furber, 2021; Yan et al., 2024).

This review proposes a unified seven-component taxonomy for DNN and SNN inference on edge hardware:


$$E_{i\,n\,f\,e\,r\,e\,n\,c\,e}=E_{c\,o\,m\,p\,u\,t\,e}+E_{m\,e\,m\,o\,r\,y}+E_{s\,t\,a\,t\,e}+E_{t\,e\,m\,p\,o\,r\,a\,l}+$$

$$E_{a\,c\,t\,i\,v\,a\,t\,i\,o\,n}+E_{l\,e\,a\,k\,a\,g\,e}+E_{c\,l\,o\,c\,k}$$

Each component is directly associated with a distinct source of hardware energy consumption, thereby linking algorithmic operations to CMOS energy costs. While prior studies focus on computation and memory, fewer explicitly address state maintenance, temporal processing, activation generation, and clock overheads (Dampfhoffer et al., 2023; Ferreira et al., 2025). Integrating these dimensions, the taxonomy provides a structured basis for analyzing inference energy trade-offs across platforms.

*E*_*compute*_ captures arithmetic energy (MAC in DNNs, AC in SNNs); *E*_*memory*_ accounts for weight/activation transfers across the memory hierarchy; *E*_*state*_ reflects state maintenance costs–output activations in DNNs, extended to per-neuron membrane potentials in SNNs (Dampfhoffer et al., 2023; Rathi et al., 2023); *E*_*temporal*_ quantifies multi-timestep processing penalties, absent in single-pass DNN inference but inherent to SNNs where energy scales with timesteps T (Yan et al., 2024); *E*_*activation*_ corresponds to non-linear function evaluation–computationally significant in DNNs (ReLU, Softmax) but reduced to binary thresholding in SNNs (Li et al., 2024); *E*_*leakage*_ represents static dissipation integrated over inference duration, relevant in always-on applications (Chundi et al., 2021); and *E*_*clock*_ quantifies synchronous clock distribution energy–representing 25%–50% of chip power in DNN accelerators (Xiao et al., 2021), largely eliminated in event-driven neuromorphic architectures.

The seven energy components can be expressed using analytical models based on hardware operation counts and technology-dependent unit energy costs:


$${\text{E}}_{\mathrm{compute}}=\sum \left({\text{N}}_{\mathrm{MAC}}\cdot {\text{E}}_{\mathrm{MAC}}\right)+\sum \left({\text{N}}_{\mathrm{AC}}\cdot {\text{E}}_{\mathrm{AC}}\right)$$ (1)

$${\text{E}}_{\mathrm{memory}}=\Sigma_{i}\,{\text{N}}_{\mathrm{access},i}\cdot {\text{E}}_{\mathrm{access},i}$$ (2)

$${\text{E}}_{\mathrm{activation}}={\text{N}}_{\mathrm{ops}\,\_\,\mathrm{NL}}\cdot {\text{E}}_{\mathrm{ALU}\,\_\,\mathrm{approx}}$$ (3)

$${\text{E}}_{\mathrm{leakage}}={\text{I}}_{\mathrm{leakage}}\cdot {\text{V}}_{\mathrm{DD}}\cdot {\text{T}}_{\mathrm{latency}}$$ (4)

$${\text{E}}_{\mathrm{clock}}=\alpha_{\mathrm{clk}}\cdot {\text{C}}_{\mathrm{clk}}\cdot {\text{V}}_{\mathrm{DD}}^{2}\cdot {\text{f}}_{\mathrm{clk}}\cdot {\text{T}}_{\mathrm{latency}}$$ (5)

$${\text{E}}_{\text{temporal}}\approx \left(T-1\right)\cdot {\text{E}}_{\text{step}}$$ (6)

$${\text{E}}_{\mathrm{state}}={\text{N}}_{\mathrm{neur}}\cdot T\cdot \left({\text{E}}_{\mathrm{Rstate}}+{\text{E}}_{\mathrm{Wstate}}+{\text{E}}_{\mathrm{comp}}\right)$$ (7)

Here, N denotes operation or transfer counts, *E*_*(.)*_ the corresponding unit energy costs, *VDD* the supply voltage, *T* the algorithmic timestep count, and *T*_*latency*_ the inference latency. The clock-network parameters α_*clk*_, *C*_*clk*_, and *f*_*clk*_ represent the switching activity factor, effective switched capacitance, and operating frequency of the clock distribution network, respectively. *E*_*step*_, *E*_*Rstate*_, *E*_*Wstate*_, and *E*_*comp*_ denote the energy of one additional timestep, state read, state write, and state update, respectively.

Though presented additively, these components are highly coupled; algorithmic changes trigger cascading trade-offs where, for instance, temporal processing multiplies memory traffic and sparsity modulates computation. This coupling persists throughout the following sections.

## 3 DNN inference energy components

In conventional DNN inference, the fundamental primitive is the multiply–accumulate (MAC) operation, aggregating weighted inputs before nonlinear activation. Energy profiles are governed by cumulative operation costs and hardware characteristics (Li et al., 2024; Ngo et al., 2025).

*E*_*compute*_ scales with MAC count–determined by model depth, layer width, and kernel dimensions–and unit arithmetic cost *E*_*MAC*_ (Equation 1), where $E_{M\,A\,C}=\alpha \times C\times V_{D\,D}^{2}$ (α = switching activity factor). Quantization (e.g., 32-bit to 8-bit) and structured pruning reduce both unit operation energy and MAC counts across MCUs, FPGAs, and ASICs (Balaskas et al., 2024; Ngo et al., 2025).

*E*_*memory*_ dominates DNN inference, reflecting Horowitz (2014) hierarchical cost model (Equation 2), where N_*access*,i_ is data transferred across memory level i and *E*_*access,i*_ its unit energy. Model weights account for most traffic, particularly in fully connected and large convolutional layers that exceed on-chip SRAM capacity, thereby requiring off-chip DRAM accesses. NVIDIA platform experiments show memory-bound layers consume disproportionate energy across precisions (Lahmer et al., 2022; Yoon et al., 2025). Yoon et al. (2025) report memory-computation balance depends on batch size and layer type: memory dominates at small batches; computation dominates in convolutional workloads with larger batches.

*E*_*activation*_ (Equation 3) introduces measurable overhead, particularly for transcendental and division operations (e.g., Sigmoid, Softmax, GELU). Li et al. (2024) show these functions are decomposed into basic arithmetic operations, often using iterative approximations such as Newton–Raphson, on MCUs without floating-point units. Li et al.’s (2024) Transistor Operations (TOs) model estimates activations account for 5%–15% of computational complexity depending on the architecture, a contribution systematically underestimated by FLOP-based metrics focused on linear MACs.

*E*_*leakage*_ and *E*_*clock*_ represent latency-dependent overheads scaling with execution time rather than computational activity. Leakage energy is modeled by (Equation 4), (Ng et al., 2022). In synchronous DNN accelerators, clock networks consume 25%–50% of total power (Equation 5), while leakage accounts for a fixed floor–approximately 122 μW in 65 nm architectures–dominating lightweight workloads (Xiao et al., 2021). As MLPerf Tiny benchmarks show power spanning microwatts to watts (Banbury et al., 2021), reducing execution latency and tuning voltage (Bartoli et al., 2025) is as critical as minimizing arithmetic operations for energy-efficient edge inference.

*E*_*state*_ and *E*_*temporal*_ are absent in feedforward networks (CNNs, MLPs), where inference is stateless without temporal dependencies (Abillama et al., 2025; Ferreira et al., 2025).

These emerge in stateful architectures. In RNNs and LSTMs, *E*_*state*_ captures maintaining hidden representations across timesteps (Rathi et al., 2023; Yik et al., 2025), introducing explicit *E*_*temporal*_ for sequential processing (Equation 6).

This amplifies in Transformers and LLMs through Key–Value cache management (Yoon et al., 2025). For Llama-7B, KV cache energy increases from 12% (batch size 1) to 60.2% (batch size 16) (Yoon et al., 2025). Impact grows with sequence length as cache scales quadratically, shifting from compute-dominated to state- and memory-driven regimes (Yoon et al., 2025).

## 4 SNN inference energy components

In SNNs, information is encoded in sparse binary spike trains propagated over discrete time steps. The fundamental primitive is the accumulate (AC) operation rather than multiply-accumulate. Each neuron integrates weighted input spikes and fires only when a threshold is crossed, yielding an event-driven energy profile distinct from feedforward DNNs (Davidson and Furber, 2021; Dampfhoffer et al., 2023; Rathi et al., 2023).

*E*_*compute*_ is modeled as *E*_*compute*_=*E*_*spike*_×*N*_*spikes*_ (Equation 1), where *E*_*spike*_ represents energy per spike (10–50 pJ on modern neuromorphic hardware), and *N*_*spikes*_ is the total number of spikes generated during inference (Imanov et al., 2026). Operation count scales with spike rate rather than network dimensionality (Dampfhoffer et al., 2023; Yan et al., 2024). Activity regularization during training can reduce spike count and synaptic operations by over 90% (Narduzzi et al., 2022).

*E*_*compute*_ benefits from two effects. First, event-driven SNNs trigger synaptic operations only upon spike arrival. Second, replacing MAC with AC operations eliminates power-hungry multipliers; in 22 nm TSMC, 32-bit addition requires approximately 60 fJ, one-fifth of the 293 fJ consumed by equivalent MACs (Davidson and Furber, 2021; Dampfhoffer et al., 2023).

*E*_*memory*_ is modulated by sparsity, as weight accesses are triggered only by incoming spikes; however, repeated weight accesses at each time step partially offset this advantage (Dampfhoffer et al., 2023; Yan et al., 2024).

*E*_*state*_ results from systematic requirements to access and update membrane potentials at every timestep (T). This State-maintenance overhead, often overlooked in hardware-agnostic analyses, scales linearly with the number of timesteps (T). Dampfhoffer et al. (2023) formulate this with (Equation 7). Intel Loihi 2 measurements reveal neuron updates account for 23% of total energy per inference (Imanov et al., 2026). While synaptic operations benefit from event-driven sparsity, *E*_*state*_ remains an obligatory architectural tax.

*E*_*temporal*_ distinguishes SNN energy profiles from single-pass execution. Total inference energy scales approximately linearly with discrete timesteps (T) required for target accuracy, as computation and memory accesses repeat at every timestep (Dampfhoffer et al., 2023). *E*_*temporal*_ captures the cost of multi-timestep execution and approximately scales with the number of additional timesteps (Equation 6), where increasing temporal resolution mitigates discretization errors at the expense of cumulative execution overhead (Rathi et al., 2023). Specifically, when T > 5, the average spike rate must remain below 5.7% for SNNs to outperform equivalent quantized DNNs (Yan et al., 2024). Otherwise, repeated memory accesses outweigh event-driven computation benefits.

*E*_*activation*_ is substantially reduced versus DNNs, as non-linear thresholding requires only simple comparison rather than floating-point evaluation (Dampfhoffer et al., 2023).

*E*_*leakage*_ is determined by fabrication technology, operating voltage, and total inference duration *T*_*latency*_ (Equation 4), (Ng et al., 2022). SNNs amplify these impacts due to their multi-timestep nature, extending static power dissipation (Yan et al., 2024; Abillama et al., 2025). Empirical 65 nm CMOS data show an ultra-low-power SNN classifier dissipates approximately 75 nW at 0.52 V without input, with chip leakage ranging from 0.02 to 1 μW across −20 °C to 65 °C and 0.5–0.65 V (Chundi et al., 2021). This cumulative overhead severely penalizes ANN-to-SNN conversion models, which typically require 100–1000 timesteps for competitive accuracy (Imanov et al., 2026). In high-latency regimes, persistent leakage dominates the budget, negating computational advantages of event-driven spike sparsity (Yan et al., 2024; Abillama et al., 2025).

*E*_*clock*_ constitutes critical overhead determined by hardware synchronization strategy (Equation 5), representing energy to distribute timing signals triggering sequential neuron state updates (Chundi et al., 2021; Carpegna et al., 2022). In traditional synchronous SNNs, the membrane potential of every neuron is updated each cycle regardless of presynaptic activity (Carpegna et al., 2022; Yan et al., 2024). The Spiker SNN accelerator on Xilinx Artix-7 FPGA consumes 13 mJ per image for MNIST when updating 400 parallel neurons (Carpegna et al., 2022).

For energy savings in resource-constrained applications, spike-driven clock-gating dynamically disables local timing signals until a spike is detected, reducing hidden neuron power by up to 74.6% versus free-running designs (Chundi et al., 2021). Alternatively, asynchronous architectures like Intel Loihi eliminate the global clock tree through localized handshake protocols, ensuring synchronization energy is consumed strictly when and where a spike is processed (Chundi et al., 2021; Deng et al., 2025).

## 5 Comparative architectural analysis

Using the seven-component framework from Section “2 Physical foundations and energy taxonomy,” we compare DNN and SNN energy profiles to identify when architectural advantages emerge.

Regarding *E*_*compute*_, the computational advantage of SNNs stems from eliminating the multiplication stage inherent to multiply–accumulate (MAC) operations, relying primarily on accumulation (AC), together with the intrinsic sparsity of spike activity. This substitution theoretically reduces per-operation energy by approximately fivefold in comparable CMOS technology (Davidson and Furber, 2021). However, Dampfhoffer et al. (2023) demonstrate through rigorous hardware-aware modeling that this advantage materializes only at very high spike sparsity–specifically fewer than 0.15–1.38 spikes per synapse per inference–a regime that is not guaranteed in practical deployments and is strongly architecture- and task-dependent (Ferreira et al., 2025).

Regarding *E*_*memory*_, both paradigms share the same physical cost hierarchy, and the SNN advantage is conditional upon spike sparsity reducing effective weight access frequency. (Yoon et al., 2025) confirm that memory energy dominates in both DNN and SNN inference at low batch sizes, and that repeated weight accesses across time steps in SNNs can partially offset the sparsity-driven reduction in memory traffic–a finding corroborated by the analytical framework of Yan et al. (2024).

*E*_*state*_ and *E*_*temporal*_ are the most distinctive components of the SNN energy profile, as they constitute inherent overheads absent in standard feedforward DNN inference. Maintaining membrane potentials introduces mandatory per-neuron, per-timestep memory access cost that scales with network dimensionality and temporal depth. Furthermore, the linear scaling of total energy with the number of timesteps T establishes a fundamental accuracy-energy tradeoff–where the temporal dimension acts as a direct multiplier on computation and memory terms–a bottleneck with no direct equivalent in the DNN paradigm (Dampfhoffer et al., 2023; Abillama et al., 2025).

Conversely, *E*_*activation*_ favors SNNs, where threshold-based spike generation replaces the floating-point non-linear evaluations required in DNNs, a distinction that becomes particularly significant on microcontrollers (MCUs) lacking dedicated hardware for transcendental functions, where these complex activations must often be decomposed into multiple basic ALU operations or simulated using lookup tables (LUTs) (Li et al., 2024; Roy et al., 2025).

The baseline of *E*_*clock*_ and *E*_*leakage*_ is dictated by the hardware substrate rather than the paradigm. On asynchronous processors like Intel Loihi, event-driven execution eliminates global clock overhead *E*_*clock*_, achieving 27× lower power than the conventional NCS2 DNN accelerator, though higher latency restricts the net energy savings to 5× (Chandarana et al., 2022). Conversely, hybrid platforms like IBM TrueNorth retain synchronous cores, while standard synchronous MCUs/FPGAs diminish SNN clock advantages entirely. Furthermore, reducing static leakage *E*_*leakage*_ requires spike-driven power-gating rather than pure asynchrony, disproportionately penalizing high-latency, multi-timestep SNN conversions compared to single-pass DNNs (Deng et al., 2025).

Taken together, these observations establish that the energy efficiency of SNNs relative to DNNs is neither universal nor unconditional, but is jointly determined by spike sparsity, the number of time steps, the hardware substrate, and the specific energy components considered. This multi-dimensional dependency underscores the necessity of component-level analysis–rather than aggregate metrics–for principled hardware-software co-design decisions in edge inference systems.

## 6 Discussion and conclusion

**FIGURE 1:**
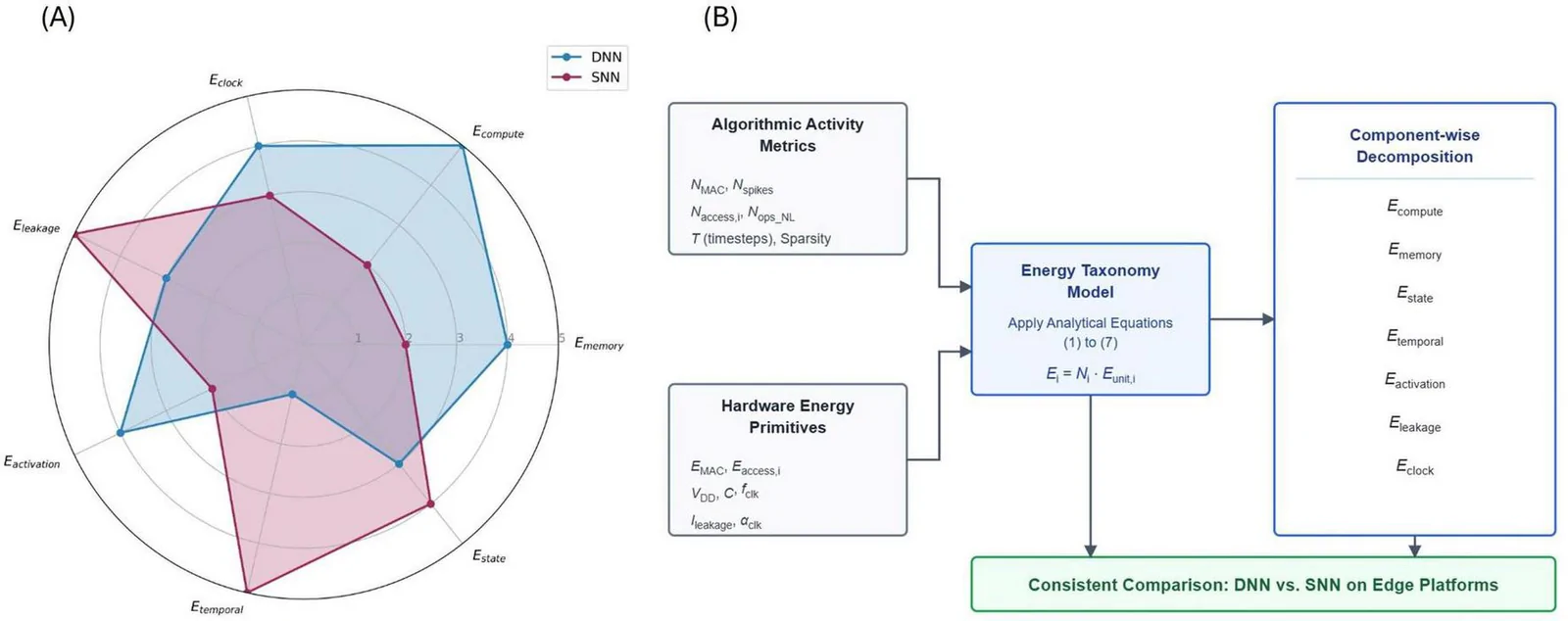
Proposed hardware-grounded energy taxonomy for comparing DNN and SNN inference. **(A)** Conceptual comparison of the relative contributions of the seven energy components in DNN and SNN inference. The radar chart is intended to summarize general trends reported in the literature rather than provide quantitative measurements. **(B)** Illustrative workflow showing how the proposed taxonomy can be applied by combining algorithmic activity metrics with hardware energy primitives to obtain a component-wise decomposition of inference energy and enable consistent comparisons across edge platforms.

By synthesizing the seven energy components identified across the reviewed studies, this review highlights that the energy efficiency of neural networks, whether DNNs or SNNs, remains highly conditional rather than intrinsic. As summarized in Table 1, these energy components are distributed across the literature, with individual studies typically focusing on specific aspects of inference energy rather than considering the complete energy profile. Organizing these components into a unified taxonomy provides a structured view of energy contributions across neural architectures. The resulting analysis highlights that energy consumption is distributed differently across DNN and SNN architectures, where computation, memory, temporal processing, and state-related costs contribute differently, as illustrated in Figure 1A. A more accurate interpretation of inference energy therefore requires a systematic hardware-aware evaluation process, following a general methodological workflow such as the one summarized in Figure 1B. This perspective helps researchers adapt evaluation procedures for specific objectives, including energy optimization, latency reduction, accuracy preservation, or hardware efficiency.

**TABLE 1:** Comparison with existing energy modeling frameworks.

Approach/framework	E_compute_	E_memory_	E_state_	E_temporal_	E_activation_	E_leakage_	E_clock_	Comparison DNN/SNN
Davidson and Furber, 2021	✓	✓	✓	✓	X	✓	X	✓
Xiao et al., 2021	✓	✓	X	X	X	✓	✓	X
Narduzzi et al., 2022	✓	X	X	X	✓	X	X	✓
Dampfhoffer et al., 2023	✓	✓	✓	✓	X	X	X	✓
Rathi et al., 2023	✓	✓	✓	✓	X	X	X	✓
Yan et al., 2023	✓	✓	X	X	X	X	✓	X
Balaskas et al., 2024	✓	✓	X	X	✓	✓	X	X
Carpegna et al., 2022	✓	✓	✓	✓	✓	X	✓	✓
Li et al., 2024	✓	✓	X	X	✓	X	X	X
Yan et al., 2024	✓	✓	✓	✓	✓	✓	X	✓
Abillama et al., 2025	✓	✓	✓	✓	X	X	X	✓
Deng et al., 2025	✓	✓	✓	✓	✓	X	✓	✓
Ngo et al., 2025	✓	✓	X	X	X	X	X	X
Roy et al., 2025	✓	✓	✓	✓	✓	✓	X	✓
Yik et al., 2025	✓	✓	✓	✓	✓	✓	X	✓
Yoon et al., 2025	✓	✓	X	X	✓	X	X	X
Imanov et al., 2026	✓	✓	✓	✓	X	✓	X	✓
Proposed	✓	✓	✓	✓	✓	✓	✓	✓

✓: The component is explicitly modeled, measured, or accounted for. X: The factor is omitted or designated as out of scope.

To preserve a rigorous comparison of neural-core efficiency, the proposed taxonomy intentionally excludes secondary energy sources. Routing energy reported by Imanov et al. (2026) was omitted because it is specific to multi-core architectures such as Intel Loihi 2, where it arises from core partitioning and Network-on-Chip communication. Nevertheless, this component should be considered for such deployments, as it may contribute up to 15% of total inference energy (Imanov et al., 2026). Mixed-signal data conversion (ADCs/DACs) was likewise excluded because, although it can exceed 50% of the energy budget in analogue Compute-in-Memory (CIM) systems, it is absent from the purely digital architectures considered here (Roy et al., 2025). Sensor pre-processing and feature extraction were also treated as external system-level costs. Following established edge benchmarking (Banbury et al., 2021), excluding these workloads enables unbiased comparison of hardware-algorithm efficiency.

Several research gaps follow from this analysis. First, the omission of *E*_*state*_ and *E*_*temporal*_ in many SNN energy benchmarks introduces a methodological inconsistency that biases comparisons with DNNs. Future evaluations should therefore adopt component-level reporting, as initiated by MLPerf Tiny (Banbury et al., 2021) and formalized by NeuroBench (Yik et al., 2025). Second, because *E*_*leakage*_ and *E*_*clock*_ are hardware-dependent, efficiency results obtained on neuromorphic processors such as Intel Loihi cannot be directly transferred to FPGA or MCU implementations, despite frequent assumptions to the contrary (Davidson and Furber, 2021; Chandarana et al., 2022; Deng et al., 2025). Third, the hardware energy overhead associated with the training phase demands careful consideration, because it affects the feasibility of edge applications.

Future work should prioritize hardware-software co-design exploiting activity regularization and improved temporal coding to mitigate*E*_*temporal*_ and *E*_*state*_ without compromising accuracy. In-memory computing combining CMOS logic with resistive memory technologies also offers opportunities to reduce both *E*_*memory*_ and *E*_*compute*_. Extending component-level analysis to recurrent DNNs, including LSTMs and transformer models with key-value caching, would further broaden the proposed taxonomy.

In conclusion, this review introduces a hardware-grounded, paradigm-neutral taxonomy of seven energy components for neural-network inference on edge hardware and illustrates its relevance for systematic comparisons between DNNs and SNNs. Rather than identifying a superior paradigm, the framework enables rigorous, application-dependent evaluation under explicit hardware constraints.
